# Specific trophoblast transcripts transferred by extracellular vesicles affect gene expression in endometrial epithelial cells and may have a role in embryo-maternal crosstalk

**DOI:** 10.1186/s12964-019-0448-x

**Published:** 2019-11-14

**Authors:** Masoumeh Es-Haghi, Kasun Godakumara, Annika Häling, Freddy Lättekivi, Arina Lavrits, Janeli Viil, Aneta Andronowska, Tamer Nafee, Victoria James, Ülle Jaakma, Andres Salumets, Alireza Fazeli

**Affiliations:** 1 Department of Pathophysiology, Institute of Biomedicine and Translational Medicine, Faculty of Medicine, Tartu University, Tartu, Estonia; 2 Department of Hormonal Action Mechanisms, Institute of Animal Reproduction and Food Research Polish Academy of Sciences, Olsztyn, Poland; 3 Academic unit of reproductive and developmental medicine, Department of Oncology and Metabolism, University of Sheffield, Sheffield, UK; 4 School of Veterinary Medicine and Science, University of Nottingham, Nottingham, LE12 5RD UK; 5 Institute of Veterinary Medicine and Animal Sciences, Estonian University of Life Sciences, Fr. R. Kreutzwaldi 1, 51006 Tartu, Estonia; 6 Department of Obstetrics and Gynecology, Institute of Clinical Medicine, University of Tartu, Tartu, Estonia; 7 Competence Centre on Health Technologies, Tartu, Estonia; 8 Department of Biomedicine, Institute of Biomedicine and Translational Medicine, University of Tartu, Tartu, Estonia; 9 Department of Obstetrics and Gynaecology, University of Helsinki and Helsinki University Hospital, Helsinki, Finland

**Keywords:** Embryo-maternal communication, Extracellular vesicles, Non-coding RNA

## Abstract

**Background:**

Successful establishment of pregnancy hinges on appropriate communication between the embryo and the uterus prior to implantation, but the nature of this communication remains poorly understood. Here, we tested the hypothesis that the endometrium is receptive to embryo-derived signals in the form of RNA.

**Methods:**

We have utilized a non-contact co culture system to simulate the conditions of pre implantation environment of the uterus. We bioorthogonally tagged embryonic RNA and tracked the transferred transcripts to endometrium. Transferred transcripts were separated from endometrial transcripts and sequenced. Changes in endometrial transcripts were quantified using quantitative PCR.

**Results:**

We show that three specific transcripts are transferred to endometrial cells. We subsequently demonstrate a role of extracellular vesicles (EVs) in this process, as EVs obtained from cultured trophoblast spheroids incubated with endometrial cells induced down-regulation of all the three identified transcripts in endometrial cells.

Finally, we show that EVs/nanoparticles captured from conditioned culture media of viable embryos as opposed to degenerating embryos induce *ZNF81* down-regulation in endometrial cells, hinting at the functional importance of this intercellular communication.

**Conclusion:**

Ultimately, our findings demonstrate the existence of an RNA-based communication which may be of critical importance for the establishment of pregnancy.

## Plain English summery

The phenomenon of an embryo attaching to the mother has fascinated scientists for generations. The embryo is essentially an outside presence in the uterus because only one half of its genetic information is originating from the mother. Why doesn’t the mother’s system refuse the attachment? One possibility would be a communication between the embryo and the mother before any attachment, convincing the mothers cells that the embryo is not a threat. Using this general theme, we developed a hypothesis that if a communication takes place, it could be in the form of RNA exchange.

We used an artificial trophoblast (outermost later of the embryo) to represent an embryo just about to implant, and endometrial cells (cells lining the inner wall of uterus) as the mothers’ cells for this experiment. We labelled the RNA in the embryo cells and put embryo and mothers’ cells together to see if any communication would take place. We were able to capture the labelled RNA inside the mother’s cells thus confirming that communication between embryo and the mother occurs and it uses RNA as the medium.

It was also apparent by the way the transport took place, that RNA is being transferred between the cells packaged in extracellular vesicles, the cargo ships of the cell world. We believe that these observations could be a stepping stone in the path of understanding the first communication between a baby and the mother.

## Background

The development of the mammalian embryo into a fully-fledged organism depends critically on its successful implantation into the uterine wall. However, as a non-self-entity, the embryo must avoid rejection by the mother’s immune system, necessitating an intricate set of negotiations before implantation can occur. Thus, the interaction between the fertilised embryo and the maternal tract arguably represents the most important diplomatic process in placental mammals. Despite this, very little is known regarding the language in which these negotiations are carried out.

That the female reproductive tract is able to detect and respond to the presence of gametes and embryos is well established and evident in the transcriptomic and proteomic profiles of the oviduct/fallopian tube and endometrial cells [1–4], suggesting that some form of signal is transmitted by the embryo. While such signals may exist in a variety of forms, several lines of evidence are pointing to the exchange of noncoding RNA (ncRNA) from embryo to mother [5] and vice versa [6] as a mean of communication leading to alterations of transcriptomic and epigenomic profiles of the maternal tract.

Recent investigations have pointed to the exchange of different forms of ncRNA as a major component of cell-to-cell communication [7–9]. These exchanges seems to be partly mediated through extracellular vesicles (EV) [10]. The term EV describes a membrane bound particle with a diameter of 40 to 1000 nm [11]. Subdivisions of EVs such as exosomes (40 - 100 nm) are termed according to their size and biogenesis [12]. EVs carry DNA, RNA and proteins and thus can facilitate cell-to-cell communication via the exchange of these molecules [13]. Different forms of RNA have been described as cargo of EVs, i.e. mRNA, ncRNA such as microRNA (miRNA) and long non-coding RNA (lncRNA) [10].

MiRNAs are small (∼22 nucleotides) non-coding single stranded RNAs which are master post-transcriptional regulators of gene expression. Over 2000 miRNAs have been discovered in the human genome, which collectively regulate over a third of the genes in the human genome [14]. LncRNA are defined as autonomously transcribed RNA with more than 200 nucleotides. Long intergenic/intervening RNA (lincRNA) are lncRNA which do not overlap protein coding genes [15]. There are over 30,000 lncRNAs discovered and annotated, approximately half of which are lincRNA [16]. There are over 150 lincRNA with a described putative function. Generally, the function of lincRNA is to modify gene expression by directly affecting nuclear architecture [15]. Expressions of many lincRNA are known to be altered in several types of cancers including endometrial carcinoma [17, 18] as well as in polycystic ovary syndrome [19]. LincRNA LINC473 is reported to be significantly involved in decidualisation by regulating some crucial decidual factors and WNT4 [20].

To best of our knowledge there are currently only two studies that have indicated the uptake of embryonic ncRNA by the endometrial cells [5] and vice versa the uptake of the maternal ncRNA by the embryos [6]. However, both of these studies focused on miRNA, and the role of other ncRNAs in maternal-embryo communication has not been addressed. Hence, to fully understand the extent of communication between mother and embryo it is important to investigate the potential of other RNA species exchanged between the embryo and the mother. In the current investigation, we tracked and captured both coding and ncRNA exchanged in cell-cell communication model using a genetic labelling system based on copper (I)-catalysed cycloaddition reaction, also known as bioorthogonal click chemistry [21]. Bioorthogonal tagging of metabolites (such as nucleic acids, proteins, glycans and lipids) uniquely enables tracking the tagged substance in vivo and in vitro [22–24], while not disrupting other physiological processes. During neurogenesis, for instance, it is possible to visualize bioorthogonally labelled RNA as it spreads over dendron cells using nascent RNA synthesis in presence of 5-ethynyl uridine (EU) [25]. Application of a similar EU-RNA labelling system in the present study led to the discovery of transcripts transferred from trophoblast to endometrial cells. Given the well-recognized ethical and technical limitations associated with the study of human embryo-endometrial dialogue in vivo [26], we used an established human in vitro implantation model using RL95–2, a human epithelial cell line derived from a moderately differentiated endometrial adenocarcinoma [27] that exhibits pronounced adhesiveness to trophoblast-derived JAr cells [28]. Although JAr and RL 95–2 cells are not perfectly similar to the trophoblast and receptive endometrium, there is evidence to infer a significant similarity due to the extensive use of these cell types in embryo adhesion and communication models [29–32]. We identified specific trophoblast transcripts that were transferred by EVs into endometrial RL95 cells, leading to the down-regulation of the same transcripts in the co-cultured recipient endometrial cells. Furthermore, EVs/nanoparticles captured from conditioned culture media of viable human embryo down-regulated the expression of at least one of the transcripts in the RL95 cells. Interestingly, co-culture of EVs/nanoparticles obtained from the conditioned culture media of degenerating human in vitro fertilization (IVF) embryos did not alter the expression of the particular endogenous transcript in RL95 cells. We suggest that these intriguing findings represent the first steps towards deciphering the ‘spoken language’ between the embryo and the mother at early stages of conception.

## Materials and methods

### Cell culture and spheroid formation

The human endometrial adenosquamous carcinoma cell line (RL95–2) was obtained from American Type Culture Collection (ATCC CRL-1671, Teddington, UK). RL95–2 was cultured in Dulbecco’s Modified Eagles Medium (DMEM 12-604F, Lonza, Verviers, Belgium) supplemented with 1% Penicillin/Streptomycin (P/S, Gibco™ 15140122, Bleiswijk, Netherlands), 5 μg/ml Insulin (human recombinant insulin, Gibco, Invitrogen, Denmark), 1% L-glutamine (Sigma, 59202C, Saint Louis, USA) and 10% fetal bovine serum (Gibco™, 10500064) at 37 °C in 5% CO_2_ atmosphere.

The human choriocarcinoma cell line (JAr) from the first trimester trophoblasts was acquired from ATCC (HTB-144™, Teddington, UK). JAr cells were cultured in a T75 flask in RPMI 1640 media (Gibco, Scotland) supplemented with 10% FBS, 1% L-glutamine and 1% P/S at 5% CO_2_ in 37 °C. At confluency, JAr cells were washed with Dulbecco’s phosphate-buffered saline without Ca^+ 2^ and Mg^+ 2^ (DPBS, Verviers, Belgium), harvested using trypsin-EDTA (Gibco® Trypsin, New York, USA) and pelleted by centrifugation at 250 g for 5 min. 1 × 10^6^ cells/ml were cultured in 5 ml of supplemented RPMI 1640 medium in 60 mm Petri dishes at 5% CO_2_ in 37 °C. The cells were kept on a gyratory shaker (Biosan PSU-2 T, Riga, Latvia), set at 295 rotations per minute (rpm) for 18 h [33]. The viability of produced spheroids was confirmed by Live/dead® viability/cytotoxicity assay kit (Molecular Probes, Eugene, Oregon, USA), according to the manufacturer’s instructions. Briefly, a working solution was prepared with the final concentration of 2 μM and 4 μM for calcein AM (acetoxymethyl ester of calcein) and EthD-1 (ethidium homodimer-1), respectively. The working solution was added directly to spheroids and incubated at room temperature for 30 min and the viability of spheroids (majority of cells emitting green fluorescence) was confirmed with florescent microscopy. The multicellular spheroids were used to mimic trophoblast cells in vitro.

The human embryo kidney (HEK) 293 T cell line was cultured in DMEM/F-12 supplemented with 10% of heat inactivated FBS (Gibco), and 1% L-glutamine (Sigma). All cells were grown in T75 flasks at 37 °C in a 5% CO_2_ atmosphere. The media was changed every second day until confluence of the cells. One million cells were counted with a haemocytometer and cultured overnight on a gyratory shaker to form multicellular spheroids as described above.

### 5-ethynyluracil tagging of trophoblast spheroids

Produced spheroids were either used without labeling (based on the particular experimental design) or labelled with 5-ethynyl uridine (EU). For labeling, about 2 × 10^3^ spheroids were incubated in 5 ml culture media supplemented with EU at a final concentration of 0.2 mM in 60 mm pPetri dishes at 5% CO_2_ in 37 °C. The spheroids were kept on gyratory shaker (Biosan PSU-2T), set at 295 rpm for 18 h. The day after labeling, spheroids were washed by placing them in a 50 ml tube. The supernatant, including single cells and incomplete spheroids, was removed. Spheroids were re-suspended in 20 ml pre-warmed culture media and after settlement, the supernatant was removed. The washing step was repeated to remove the EU molecules from the spheroid’s environment. The labelled spheroids were prepared for co-culture system.

### Non-contact co-culture of trophoblast spheroids with endometrial cells

Endometrial cells were cultured (seeding density 1.25 × 10^6^) in each well of 6-well plate until 60% confluency. For co-incubation of trophoblast spheroids with epithelium, a 0.4 μm membrane insert was inserted in each well (Falcon® Permeable Support for 6 Well Plate with 0.4 μm Translucent High Density PET Membrane). The depth of the insert allowed the membrane to be immersed in the culture media covering the epithelial cells but not in direct contact with the cells (so-called the non-contact co-culture system). Then, approximately 2 × 10^3^ labelled spheroids were inserted on a 0.4 μm membrane insert in each well of a 6-well plate. The labelled spheroids and endometrial cells were co-incubated in serum-starved media consisted of DMEM (DMEM/F12, Verviers, Belgium v/v 1:1) supplemented with 1% L-glutamine, 1% P/S, transferrin (10 mg/ml; BioReagent, Cat. No. T8158), selenium (25 mg/L; Sigma, Cat. No. 229865), bovine serum albumin (1 mg/ml; HyClone™, Cat. No. SH30574), linoleic acid (4.7 mg/ml; Sigma, Cat. No. L1012) and insulin (5 mg/ml) for 24 h.

### Total RNA extraction and quality control

Total RNA was extracted from endometrial cell line, conditioned media and EVs by TRIzol Reagent and ethanol precipitation (TRIzol® reagent; Invitrogen). To increase the efficiency of RNA extraction, 2 μl glycogen (UltraPure™ Glycogen, Cat. no. 10814–010, Thermo Fisher Scientific, Bleiswijk, Netherlands) was added to the lysis buffer per sample. The RNA pellet was washed three times by 70% ethanol. Quality and quantity of the extracted RNA samples were analysed by Bioanalyzer Automated Electrophoresis instrument (Agilent technologies, Santa Clara, CA) using Agilent RNA 6000 Pico Kit (Agilent technologies) and Agilent Small RNA kit (Agilent technologies).

### Affinity precipitation of EU-labelled RNA

EU-labelled RNA was affinity precipitated according to the manufacturer’s instruction of Click-iT Nascent RNA capture kit (Thermo Fisher Scientific, Waltham, MA; Cat. No. C10365). Briefly, the extracted total RNA from cell lines, conditioned media and/or EVs were biotinylated in click-it reaction mixture with a final concentration of 1 mM biotin azide. The click-it reaction mixture was incubated for 30 min at room temperature while gently mixing using a gyratory shaker with 500 rpm. Biotin-azide (PEG4 carboxamide-6-azidohexanyl biotin) was attached to alkyne reactive group of the EU-labelled RNA using click chemistry. Biotinylated RNA, was incubated with 12 μl MyOne™ Streptavidin T1 magnetic Dynabeads® into Click-iT RNA binding buffer for a final volume of 74 μl. The mixture of RNA and bead was incubated in the dark at room temperature for 40 min while mixing using a gyratory shaker, 500 rpm speed to prevent the beads from settling. After biotinylated RNA binding to Dynabeads, beads were washed three times with two wash buffers that were included in the kit (pre-warmed to 65 °C), while mixing vigorously with a gyratory shaker at 700 rpm to remove the non-specifically attached RNA. After the last wash, the beads were immobilized by the DynaMag™-2 magnet and wash buffer was completely removed. Beads were re-suspended in 15 μl nuclease free water and were directly used for cDNA synthesis for sequencing and quantitative polymerase chain reaction (qPCR).

### cDNA library preparation and sequencing of captured EU-labelled RNA from endometrial cells

Ovation RNA-Seq System V2 (NuGEN technologies, San Carlos, CA, Cat.No.7102–32) was used for cDNA library synthesis. The manufacturer’s protocol was slightly modified to allow single strand cDNA to be synthesised (ssDNA) from on-bead RNA fragments. The modifications were as follows, 2 μl of First Strand Primer Mix was added to 14 μl on-bead RNA fragments and incubated for 5 min at 65 °C, followed by cooling on ice for 5 min. Then, 0.5 μl of first strand enzyme mix and 5 μl of first strand buffer mix were added to the mixture resulting in a final volume of 20 μl. The mixture was incubated at 43.5 °C for 60 min on an Eppendorf thermomixer (700–800 rpm) to prevent the beads from settling. Finally, the mixture was thermal shocked at 85 °C for 10 min and beads were rapidly immobilized by a magnet allowing the collection of cDNA from the supernatant. Ten μl of first strand cDNA was used in the double strand synthesis step. Double strand cDNA synthesis was performed according to NuGEN manufacturer’s instructions. cDNA quality was measured by High Sensitivity DNA 1000 Assay Kit (Agilent technologies). Double stranded cDNA was subsequently used for barcoded library preparation. Libraries were prepared using the AB Library Builder™ System (Thermo Fisher, Cat. No. 4477598) and Ion Xpress™ Plus Fragment Library Kit (Thermo Fisher), according to the manufacturer’s instructions. The barcoded libraries were sequenced on two Ion 540™ Chips (ThermoFisher Scientific Inc., CA, USA, Cat. No. A27766) with four libraries per chip using the Ion S5 XL sequencer (Thermo Fisher Scientific Inc).

### Differential expression analysis of RNA-seq data

The experimental methods used for detecting transferred transcripts resulted in the selective enrichment of transferred transcripts. This enrichment was quantified by conventional differential expression analysis methods since the measured effect was the alteration in the relative quantity of transcripts in one experimental group compared to another. Sequenced reads were first aligned to the hg19 human reference genome using the Torrent Mapping Alignment Program (TMAP; Thermo Fisher Scientific), using mapping algorithm map 4 with default parameters. TMAP is a sequence alignment software optimized specifically for mapping reads produced by Ion Torrent sequencing platforms. Read counts were obtained for 55,766 annotated coding and non-coding genomic elements in the hg19 human reference genome. Differential gene expression analysis of RNA-sequencing (RNA-seq) data was performed using the Generalized Linear Model (GLM) pipeline of edgeR package in R [34, 35]. The genomic elements failing to surpass counts per million (CPM) cut-off of 0.7 for at least 3 out of 4 samples in at least one of the experimental groups were omitted from further analysis. The threshold CPM ≥ 0.7 translates to 10 aligned reads per genomic element divided by the mean of total sequenced reads of all samples in millions. The differentially expressed transcripts were considered significant if the false-discovery rate (FDR) reported by edgeR was less than or equal to 0.05 (FDR ≤ 0.05). Integrative Genomics Viewer (IGV) was used to inspect the coverage of differentially expressed (enriched) transcripts.

### cDNA synthesis and qPCR analysis for quantification of EU-labelled transferred transcripts

EU-labelled RNAs from the complete conditioned media and EVs were affinity precipitated and the copy number of EU-labelled *ZNF81*, exonic-LINC00478 and intronic-LINC00478 were quantified. For cDNA synthesis of EU-labelled transferred transcripts, a mixture of random hexamer and oligo (dT) primers was used (SuperScript® VILO™ cDNA synthesis kit, 11,754 050). For EU-labelled RNA on bead, the cDNA synthesis was performed according to the Click-iT RNA Capture Kit. The primers for transferred transcripts (*ZNF81*, exonic and intronic-LINC00478) were designed by Beacon designer 8 (PREMIER Biosoft International, Palo Alto, CA) and reads sequences were used as template (Additional file 1: Table S1). For quantification of EU-labelled *ZNF81* and exonic-LINC00478, cDNA products were amplified in EvaGreen assay system (Solis BioDyne, Tartu, Estonia) with the following program: 95 °C for 15 min, followed by 40 cycles of 95 °C for 20 s, 60 °C for 20 s, and 72 °C for 20 s. For melting curve analysis, the fluorescence signals were collected continuously from 65 °C to 95 °C at 0.05 °C per second.

For quantification of EU-labelled intronic-LINC00478, the cDNA product was amplified in EvaGreen master mix, including 5% DMSO with following real-time touchdown PCR program: starting with 31 cycles of 94 °C for 20 s, the decreasing annealing temperature for 20 s, and extension of 72 °C for 20 s. The annealing temperature decreased 0.1 °C per cycle from 63.6° to 60 °C. For melting curve analysis, the fluorescence signals were collected continuously from 65 °C to 95 °C at 0.05 °C per second.

For spike-in and normalizing of candidate transferred transcripts, 100 bp from Isopenicillin N-CoA synthetase gene was used (Biomer.net company, Ulm/Donau, Germany, molecular weight: 32239 g/mol, 100 pmol/μl) (Spike-in synthetic RNA Sequence refer to the Table.1). Synthetic RNA was serially diluted 20 times. For the first serial dilution, 1 μl of synthetic RNA was added to 39 μl RNase-free water to final concentration of 2.5 μM. Serial dilutions were prepared with a dilution factor of 4x. Serial dilutions were reverse-transcribed and amplified using real-time PCR and the cycle threshold (Ct) values of dilutions were plotted against the copy number of transcript. Exponential calibration curve was fitted. In parallel, 1 μl of synthetic transcript was added to the sample during TRIzol RNA extraction and then the Ct of synthetic RNA in this sample was assayed to calculate the RNA extraction efficiency and normalizing factor [36].

**Table 1:** The table of primers and sequence information

Transcript Name	Primer Sequence (5′-3′)
ZNF81	Forward primer: TGATACAGAAGACTTGAGATT Reverse primer: TCACAAAGTATTCACATTACC
Exonic LINC00478	Forward primer: TCAAGTTCAGTGTTTGGTTAA Reverse primer: GGCAGAATCGTGAATAGC
Intronic LINC00478	Forward primer: AACAGGTCACAATGGTGGAATG Reverse primer: TGAAGCAACTGAAGATCCACAA
Beta-2-microglobulin	Forward primer: CGGGCATTCCTGAAGCTGA Reverse primer: TGGAGTACGCTGGATAGCCT
Beta-actin	Forward primer: GTGCGCCGTTCCGAAAGT Reverse primer: ATCATCCATGGTGAGCTGGCG
Synthetic RNA Spike-in (100 bp from Isopenicillin N-CoA synthetase)	Spike-in Forward primer: TACTGCATCCCGCTCTAC Spike-in Reverse primer: CGCTCATCAAGTCGTTCA Spike-in RNA sequence: UUGGGCAGAAACCGGGCCCCAACGGUGACCGCACCUACU ACUGCAUCCCGCUCUACCACGGAACGGGGGGCAUCGCGGCCAUGAACGACUUGAUGAGCGG

### Confocal laser scanning and imaging of EU-labelled RNA

The transferred EU-labelled RNAs were tracked by Alexa Fluor 488 azide (Included in Click-iT® RNA Imaging Kit; Invitrogen, C10329). After 24 h co- culture of endometrial cells with EU-labelled spheroids, the conditioned media was removed and the endometrial cells were incubated with pre-warmed cell tracker working solution for 30 min (CellTracker™ Deep Red dye; Life Technologies, C34565). After incubation the cells were washed with DPBS, fixed with 4% formaldehyde (Thermo Fisher, GmbH) and permeabilized with 0,1% Triton X-100 in PBS (AppliChem GmbH, Darmstadt, Germany). Next, the EU-labelledRNA was detected using the Click-iT® RNA Imaging Kit (Invitrogen, C10329) according to the kit protocol. Confocal laser scanning microscopy was performed using LSM510 Laser Scanning Confocal Microscope (LSM 510 Duo; Carl Zeiss Microscopy GmbH, Jena, Germany).

### EVs purification and nanoparticle tracking analysis (NTA)

Co-culture EVs were harvested from conditioned media of trophoblast spheroids/endometrial cell co-culture. Three millilitres of conditioned media from each well of 6-well plate dish was collected and 3 μl from RNase inhibitor (Solis BioDyne, Tartu, Estonia) was added to conditioned media. Conditioned media was centrifuged at 400 xg for 10 min. The supernatant was further centrifuged at 4000 g for 10 min and the supernatant was further centrifuged at 20,000 g for 15 min to get rid of cell debris and apoptotic bodies. The supernatant was filtered two times with 0.2 μm filter. To isolate EVs, filtered conditioned media was concentrated to 500 μl with Amicon® Ultra-15 centrifugal filter devices (10 kDa cut-off). EVs were isolated using size exclusion chromatography (SEC). A cross linked 4% agarose matrix of 90 μm beads were used (Sepharose 4 fast flow™, GE HealthCare Bio-Sciences AB, Uppsala, Sweden) in a 30 cm column. Fractions 7–10 (fraction size 1 ml) were collected. Fractions were concentrated using Amicon® Ultra-15 centrifugal filter devices (10 kDa cut-off). Isolated EVs were quantified using NTA (ZetaView, Particle Metrix GmbH, Inning am Ammersee, Germany). When preparing spheroid-derived EVs, conditioned media from 24 h cultures of spheroids in 60 mm dishes were used.

### Collection of human embryo conditioned culture media, EV/nanoparticles purification and characterization

Experiments with human IVF embryo conditioned culture media were carried out under the ethical approval of Research Ethics Committee of the University of Tartu, approval number 267/T-2. Human embryos were produced by IVF or intracytoplasmic sperm injection (ICSI). They were cultured individually for 17–21 h (day 1) in sequential fertilization media (Sequential Fert™, Origio, Måløv, Denmark), 48 h (day 3) in sequential cleavage stage media (Sequential Cleav™, Origio) and additionally 48 h (day 5) in sequential blastocyst stage media (Sequential Blast™, Origio). At day 3, embryos with equal size blastomeres and no fragmentation were considered as normal. At day 5, embryos with identifiable inner cell mass, trophoblast and blastocyst cavity were considered normal while embryos with degrading cells were considered as degraded. Embryo conditioned media (50 μl) was collected and subjected to low speed spin (400 x g, 2000 g). EVs/nanoparticles were isolated from the media using SEC. Namely 8–10 fractions with the volume of 1 ml were collected for further concentration in 10 kDa Amicon® Ultra-15 Centrifugal Filters (Merck Millipore, Burlington, Massachusetts, United States). Concentration of EVs/nanoparticles were measured using NTA (ZetaView).

### Western blot analysis

Purified EVs from trophoblast spheroids were precipitated by adding 200 μl of water, 400 μl of methanol and 100 μl of chloroform to 200 μl of EVs. The solution was vortexed and centrifuged 14,000 g for 5 min at room temperature. After removing the top layer, precipitated proteins were washed with 400 μl of methanol and centrifuged again. The pellets were air-dried, resuspended in 0,5% SDS and the protein concentrations were determined by Bradford assay. 30 μg of protein were heated for 5 min at 95 °C in reducing (for Apo A-I detection) or in non-reducing (for CD63, CD9 and CD81 detection) Laemmli buffer and resolved in 12% SDS-PAGE according to standard protocol. Proteins were transferred onto polyvinylidene difluoride membrane (Thermo Fisher Scientific), followed by blocking in 5% non-fat dry milk in PBS-T (0,05% Tween-20, Thermo Scientific, Michigan, USA) for 1 h at room temperature. Subsequently, membranes were incubated with the primary anti-CD63 (sc-5275, 1:1000, Santa Cruz Biotechnology Inc., Dallas, TX), anti-CD9 (MA1–80307, 1:1000, Thermo Fisher Scientific, Loughborough, UK), anti-Apo A-I (sc-376,818, 1:1000, Santa Cruz Biotechnology Inc. Dallas, TX), and anti-CD81 (555,675, 1:1000, BD Biosciences, New Jersey, USA) antibodies overnight at 4 °C in 5% milk-PBS-T solution and then with horseradish peroxidase conjugated goat anti-mouse secondary antibody (sc-516,102, 1:1000, Santa Cruz Biotechnology Inc. Dallas, TX) for 1 h at room temperature. Membranes were washed three times for 5 min in PBS-T after each incubation step. Protein bands were detected using ECL Select™ Western Blotting Detection Reagent (GE Healthcare, Buckinghamshire, UK) with ImageQuant™ RT ECL Imager (GE Healthcare, Buckinghamshire, UK).

### Electron microscopy

Suspension of EVs was deposited on formvar-carbon-coated 200 mesh cooper grids (Agar Scientific, Essex, UK) for TEM analysis according to the method described by Thery et al. 200,615. Briefly, EVs on grids were fixed in 2% paraformaldehyde (P6148, Sigma-Aldrich, Schnelldorf, Germany) and 1% glutaraldehyde (O 1909–10, Polysciences, Warrington, USA), before being contrasted in uranyl oxalate [mixture of 4% uranyl acetate (21447–25, Polysciences, Warrington, USA) and 0,15 M oxalic acid (75,688, Sigma-Aldrich, Schnelldorf, Germany)] and embedded in a mixture of methylcellulose (M6385, Sigma-Aldrich, Schnelldorf, Germany) and uranyl acetate (21447–25, Polysciences, Warrington, USA). Samples were observed with a JEM 1400 transmission electron microscope (JEOL Ltd. Tokyo, Japan) at 80 kV, and digital images were acquired with a numeric camera (Morada TEM CCD camera, Olympus, Germany).

### Statistical analysis

Data were presented as mean ± standard error of mean (SEM). In experiments that warranted statistical analysis for comparison of means, one-way ANOVA was used with appropriate post hoc analysis after testing the homogeneity with Leven’s test.

## Experimental design

### Characterization of transcripts transferred from trophoblast to endometrial cells

**Fig. 1:**
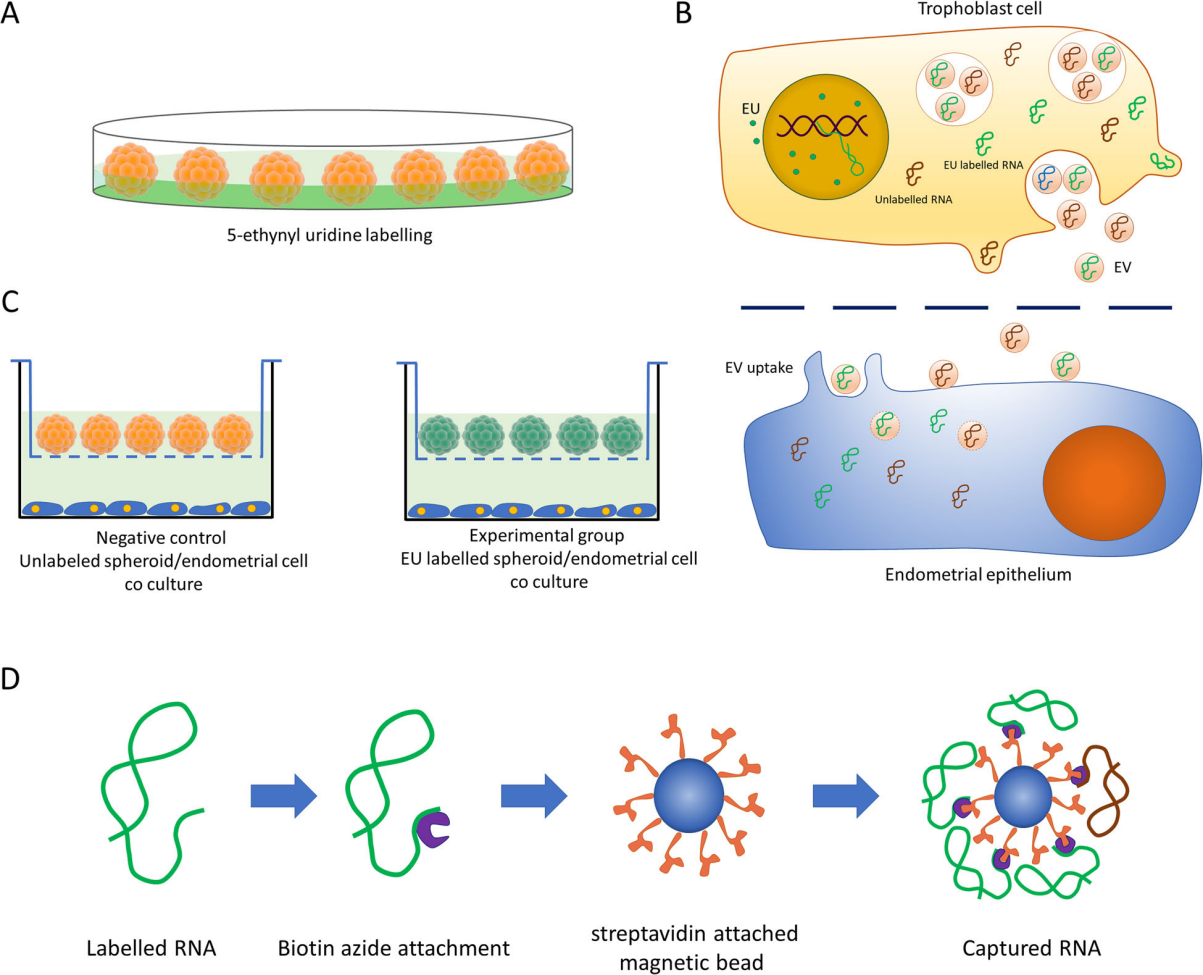
Bioorthogonal labelling strategy. **a** 5-ethynyl uridine (EU) labelling of trophoblast spheroids. Spheroids were placed in culture media supplemented with EU overnight. **b** Non-contact co-culture of trophoblast spheroids and endometrial cells. EU (green) is incorporated into nascent RNA resulting in EU labelled RNA (green). RNA is packaged into extracellular vesicles (EV) and transferred to the endometrial cells through the translucent barrier. EV containing the labelled RNA is uptaken by the endometrial cells. In the endometrial cytoplasm, RNA is released through the degrading EV membrane. **c** Experimental setup. Negative control is prepared using unlabelled trophoblast spheroids/endometrial cells. Experimental group consists of EU labelled trophoblast spheroids/Endometrial cells. **d** Affinity precipitation procedure. Labelled RNA is attached to biotin azide by click chemistry. Magnetic beads attached to streptavidin is used to selectively precipitate EU labelled RNA

To identify the RNA species that originate from trophoblast spheroids and are transferred to the endometrial cells, the trophoblast spheroids were incubated with the endometrial cells in the non-contact co-culture system as described earlier. The experimental group consisted of EU-labelled spheroids while non-EU-labelled spheroids were used as a negative control. After 24 h co-incubation, the transferred EU-labelled transcripts were affinity precipitated from the total RNA obtained from the endometrial cells. The first and second strand cDNA were synthesized and cDNA library was prepared for sequencing of the precipitated EU-labelled RNA as described earlier. Total RNA-seq was conducted with synthesized cDNA from experimental group (*n* = 4) and negative control group (*n* = 4) (Fig. 1). The bioinformatics analysis of RNA-seq data and differential expression analysis of the detected transcripts were performed to identify the putatively transferred RNA sequences. After identification of putatively transferred RNA sequences, the presence of the candidate RNA species was confirmed in the endometrial cells by qPCR.

### Identification of the route of transfer of RNA from trophoblast cells to endometrial cells

To illustrate the route of RNA transfer from trophoblast cells to endometrial cells, conditioned media was collected from the EU-labelled trophoblast spheroid/endometrial cell co-culture of 24 h (experimental group). A similar co-culture of unlabelled spheroid/endometrial cells was used as a negative control. Conditioned media from each group was divided into two similar parts by volume. One part was used for EV purification. Total EU-labelled RNA was extracted from both conditioned media and isolated EVs using affinity precipitation. Extracted RNA was quantified for the expression of transferred transcripts by qPCR.

### Visualization of transferred transcripts by confocal microscopy and Alexa Fluor 488 azide

The transferred EU-labelled RNAs were visualized in endometrial cells by Alexa Fluor 488 azide. After 24 h co-incubation of endometrial cells with EU-labelled spheroids, the endometrial cells were stained with Alexa azide and the confocal microscopy imaging was performed on both experimental and negative control group, concurrently.

### The effect of trophoblast spheroid co-culture on expression of specific RNA transcripts in endometrial cells

Approximately 1 × 10^3^ trophoblast spheroids were co-cultured with 5 × 10^5^ endometrial cells for 24 h in 12 well cell culture plates with 0.4 μm translucent inserts. Total RNA from endometrial cells were isolated and analysed for the expression of candidate transcripts by qPCR. As controls, endometrial cells co-cultured with HEK293 spheroids and untreated endometrial cells were also analysed.

### The effect of trophoblast derived EVs on expression of specific RNA transcripts in endometrial cells

To demonstrate the effects of EVs on endometrial transcripts, EVs derived from JAr cells were incubated with endometrial cells in the ratio 50:1. (2.5 × 10^7^ EVs: 5 × 10^5^ cells) (). EV number was similar to the amount of EVs produced by 1000 trophoblast spheroids in 24 h. Untreated controls were prepared with endometrial cells without EV treatment. Endometrial cells treated with similar concentrations of EVs derived from HEK293 spheroids and untrated endometrial cells were used as negative controls. After 24 h of incubation, the cells were lysed for total RNA extraction. cDNA was prepared and qPCR was performed for candidate transcripts. Beta actin and Beta-2-microglobulin were used as control genes to evaluate the behaviour of unaffected genes in endometrial cells (Additional file 1: Table S1).

### The effect of human IVF embryo-derived EVs/nanoparticles on specific RNA transcripts from endometrial cells

On day 3 post IVF, conditioned media were collected from 4 embryos that developed narmally untill day 5 and from 4 embryos that degenerated on day 5. The embryos developed until day 5 and conditioned media were again collected from 4 normal and 4 degenerated embryos. Conditioned media from each group were pooled and EVs/nanoparticles were isolated. EVs/nanoparticles were then supplemented to endometrial cells in 50:1 ratio (1 × 10^7^ EVs/nanoparticles: 2 × 10^5^ cells). Endometrial cells without EVs/nanoparticle treatment were used as negative control. After 24 h of incubation total RNA was extracted from cells, cDNA was prepared and qPCR was performed for candidate transcripts. Beta actin and beta-2-microglobulin were used as control genes.

## Results

### EU-labelled transcripts were visualized in endometrial cells by confocal microscopy

To identify possible trophoblastic RNA species that are transferred to the endometrial cells, trophoblast-derived JAr spheroids were incubated with the endometrial cells in a non-contact co-culture system. Produced spheroids were either used without labelling (based on the particular experimental design) or labelled with 5-ethynyl uridine (EU). Figure 1 depicts the overall strategy of biorthogonal labelling of trophoblast cells and capture of EU-labelled RNA in the endometrial cell.

**Fig. 2:**
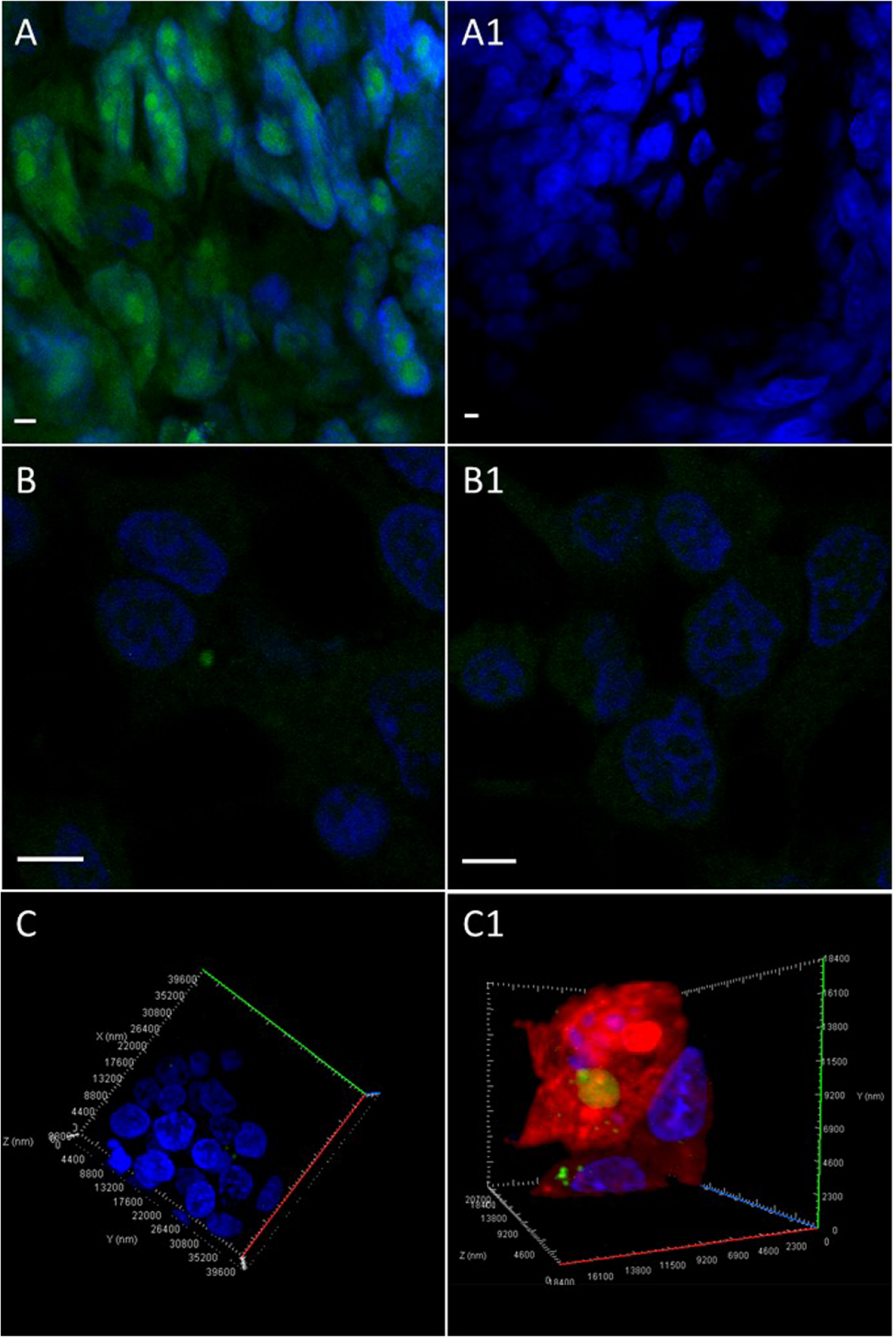
Visualization of 5-ethynyl uridine (EU)-labelled RNA in trophoblast spheroids and endometrial cells. **a** RNA in trophoblast spheroids were labelled with 5-ethynyl uridine (EU) and stained with Alexa azide. Green florescence is evidence of successful labelling. **a1** Unlabelled spheroids (negative control) did not show fluorescent signal. **b** Endometrial cells were stained with Alexa azide after 24 h incubation with labelled spheroids to visualize the transferred transcripts. Green dots in endometrial cells indicate labelled RNA transfer. **b1** Endometrial cells co-incubated with unlabelled spheroids were used as negative controls. Negative control did not exhibit any specific fluorescent signal. **c**, **c1** 3-dimentional confocal scanning of endometrial cells with cytoplasmic EU labelled RNA with and without cell tracker dye. Scale bar 4 μm

Using confocal microscopy, we observed that EU-labelled spheroids exhibited the green fluorescence signal of Alexa 488 in the nuclei and especially in the nucleoli of the spheroids, confirming the successful EU incorporation into RNA while unlabelled control spheroids showed virtually no staining (Fig. 2a, a1). When incubating endometrial cells with EU-labelled spheroids for 24 h we could detect single green fluorescent dots in the cytoplasm of the cells while the overall cytoplasmic staining was low (Fig. 2b) indicating the possible transfer of EU labelled RNA from spheroids to endometrial cells. We did not detect any similar concentrated dots with green fluorescence in the endometrial cells co-incubated with unlabelled spheroids (Fig. 2b1). The presence of EU-labelled transferred RNA in the cytoplasm of endometrial cells was confirmed by 3-dimensional confocal scanning with and without cell tracker dye (Fig. 2c, c1).

### Identification of putatively transferred transcripts from trophoblast spheroids to endometrial cells

Trophoblast spheroids with EU labelling (experimental group) were co-incubated with endometrial cells in a non-contact cell culture system to identify the transferred transcripts. Unlabelled spheroids were co-incubated with endometrial cells as a negative control. After 24 h of incubation, total RNA from endometrial cells were collected and affinity precipitated to capture EU labelled RNA. Captured RNA was used for RNA sequencing (RNA-seq).

The percentage of the EU labelled RNA recovered from the total RNA obtained from cells exposed to EU labelling was calculated to determine the efficiency of EU labelled RNA capturing procedure. In EU labelled spheroids, only 12.66% (± 1.01%) of RNA was precipitated by affinity precipitation procedures.

In endometrial cells co-incubated with labelled JAr spheroids, 2.85% (± 0.45%) of RNA was precipitated. In endometrial cells co-incubated with unlabelled JAr spheroids (negative control), 1.13% (± 0.2%) of RNA was precipitated. The results indicated that approximately 35% of the supposedly EU labelled precipitated RNA might be unlabelled and non-specifically captured by the magnetic beads.

RNA-seq yielded on average 13.5 million reads per sample with average read length of 178 base pairs. The proportion of base pairs exceeding Phred quality score of 20 (base call confidence ≥99%) was 0.81 ± 0.01 (mean of all samples ± SD). The sequencing data has been uploaded to the NCBI SRA repository (www.ncbi.nlm.nih.gov/sra) under the accession number PRJNA527834. The results of read alignment to the hg19 human reference genome varied extensively between the samples with alignment percentage ranging from 31 to 91%. This did not, however, have a major effect on the group averages, as the average alignment percentages were 51 and 55% for the experimental and control group, respectively.

**Fig. 3:**
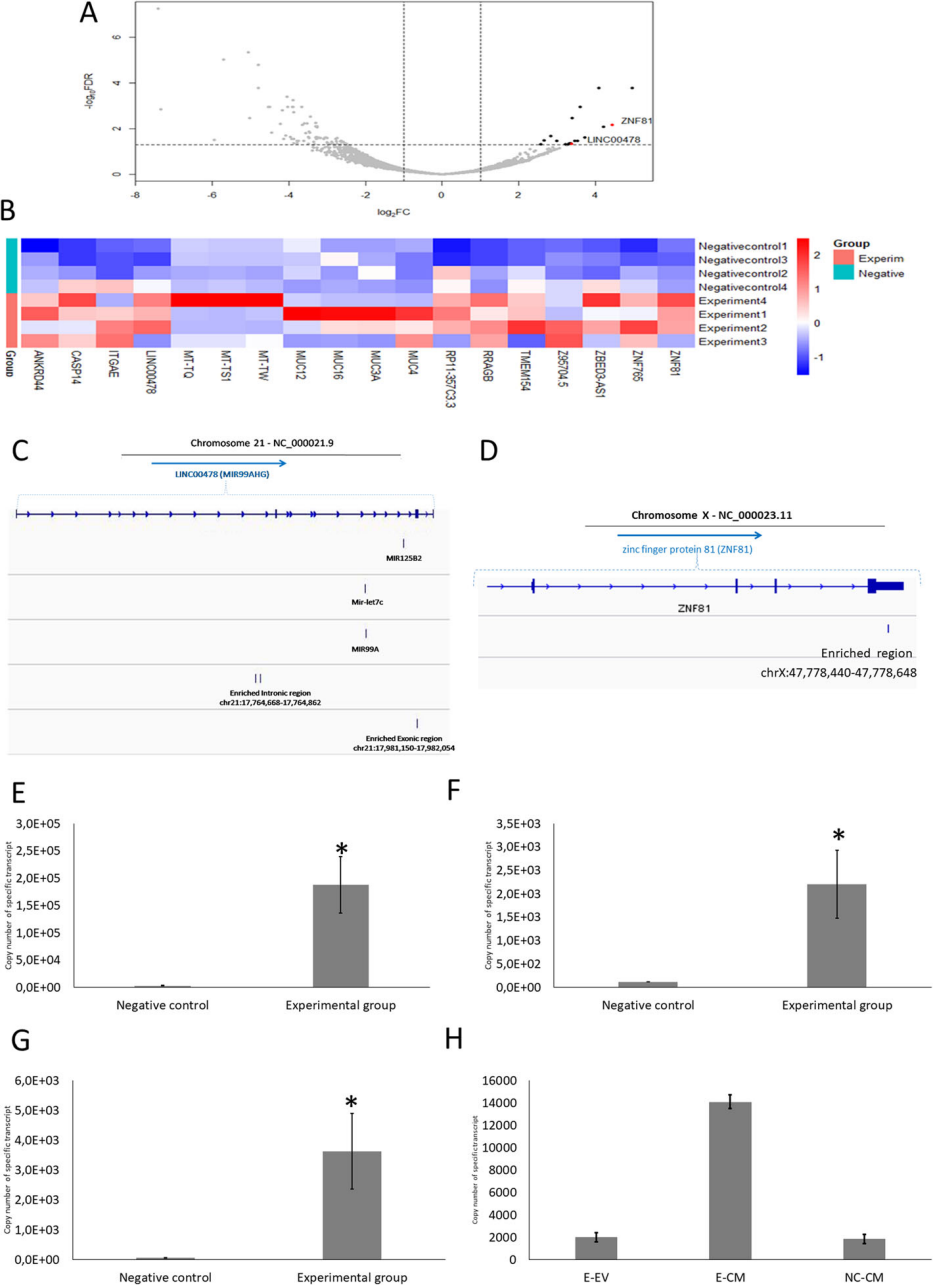
RNA sequencing of transferred 5-ethynyl uridine (EU)-labelled transcripts. **a** Volcano plot from RNA sequencing data of EU-labelled transferred transcripts affinity precipitated from endometrial cells co-incubated with EU-labelled trophoblast spheroids. RNA extracted from endometrial cells co-incubated with unlabelled spheroids were used as negative control. The rate of false discovery is plotted against fold change, demonstrating the 18 putatively transferred transcripts which were significantly enriched in experimental group (black dots). Candidate transferred transcripts were highlighted by red dots (ZNF81 and LINC00478). **b** Heatmap displaying the relative abundances of transcripts enriched in the experimental group compared to the negative control. The values presented on the heatmap are z-scores calculated based on the normalized read counts. Unsupervised hierarchical clustering of samples based on Euclidean distance calculated from presented z-scores is displayed alongside the heatmap. **c** Position of enriched intronic- LINC00478 and exonic- LINC00478 in relation to chromosome 21. **d** Position of enriched ZNF81 in relation to chromosome X. Copy number of EU-labelled (**e**) Intronic-LINC00478 (**f**) Exonic-LINC00478 and (**g**) ZNF81 were measured in endometrial cells co-incubated with EU-labelled trophoblast spheroids (Experimental group) by using qPCR and absolute quantification. Endometrial cells co-incubated with unlabelled trophoblast spheroids were used as a control (Negative control). Data is presented as mean ± SEM. (*) *p* < 0.05 vs negative control. **h** Presence of intronic-LINC00478 was observed in EU-labelled spheroid/endometrial cell co-culture conditioned media (Experimental group, E-CM) and extracted EVs (Experimental group, E-EV), and in EU-unlabelled spheroid/endometrial cell co-culture conditioned media (Negative control, NC-CM). Exonic-LINC00478 and ZNF81 were not detected in either group. Data is presented as mean ± SEM

Differential expression (DE) analysis, showed statistically significant enrichment of eighteen genomic elements in the endometrial cells. These elements were presumed to be transferred transcripts from trophoblast cells to endometrial cells (Fig. 3a, b, Additional file 1: Table S2). The alignments of individual reads to the 18 genomic elements of interest were visually inspected using Integrative Genomics Viewer (IGV), to estimate the full sequences of potentially transferred transcripts. This enabled the exclusion of genomic elements, for which the counted reads were presumed to be originating from random RNA fragments not specifically enriched but rather representing the random noise of the EU-labelled RNA capturing process.

The genomic sequences were considered to be specifically enriched when the alignment of reads originating from random RNA fragments were aligned to specific sequences and were: i) detected in at least three biological repeats out of four in the experimental group and ii) were not detected in any of the negative control samples. Only three candidate transcripts passed these stringent selection criteria: an intronic-non-coding region and an exonic-coding region, originating from LINC00478 locus of chromosome 21 (Fig. 3c) and one exonic region from *ZNF81* gene (Fig. 3d). These transcripts were selected for further analysis.

The presence of EU-labelled intronic-LINC00478 (Fig. 3e), exonic-LINC00478 (Fig. 3f) and *ZNF81* (Fig. 3g) were also confirmed in endometrial cells by qPCR after 24 h co-incubation and there was a significant difference between the experimental group and the negative control group. Sanger sequencing of qPCR products confirmed the sequences of the candidate transcripts (Additional file 1: Table S3).

### EU-labelled intronic-LINC00478 transcript was detected in conditioned co-culture media

Conditioned media was collected from EU labelled spheroid/endometrial cell co-culture (experimental group) and unlabelled spheroid/endometrial cell co-culture (negative control). Half of the conditioned media from each group was used to extract EVs. Whole RNA of the condition media and EV were extracted and subjected to affinity precipitation. Precipitated RNA was analysed for the presence of candidate transcripts using qPCR.

The presence of EU-labelled intronic-LINC00478 transcript in conditioned media was confirmed by qPCR (Fig. 3h). Copy number of this transcript was significantly higher in RNA extracted from complete conditioned media (including free RNA, RNA bound to proteins and RNA in EVs) compared to the RNA extracted from EVs. The conditioned media of the negative control also exhibited the presence of a small copy number of (7 times less than that of the experimental group) intronic-LINC00478 transcript. The presence of EU-labelled exonic-LINC00478 transcript or EU-labelled *ZNF81* transcript were not detected in conditioned media or in EVs via our qPCR assay conditions due to the low copy numbers present in the samples.

### Trophoblast spheroid derived nanoparticles were confirmed as EVs using nanoparticle tracking analysis (NTA), electron microscopy and Western blot analysis

Conditioned media from trophoblast spheroids were collected and nanoparticles were isolated using sequential centrifugation and size exclusion liquid chromatography (SEC). isolated particles were characterized using NTA, western blotting for EV specific proteins and electron microscopy.

**Fig. 4:**
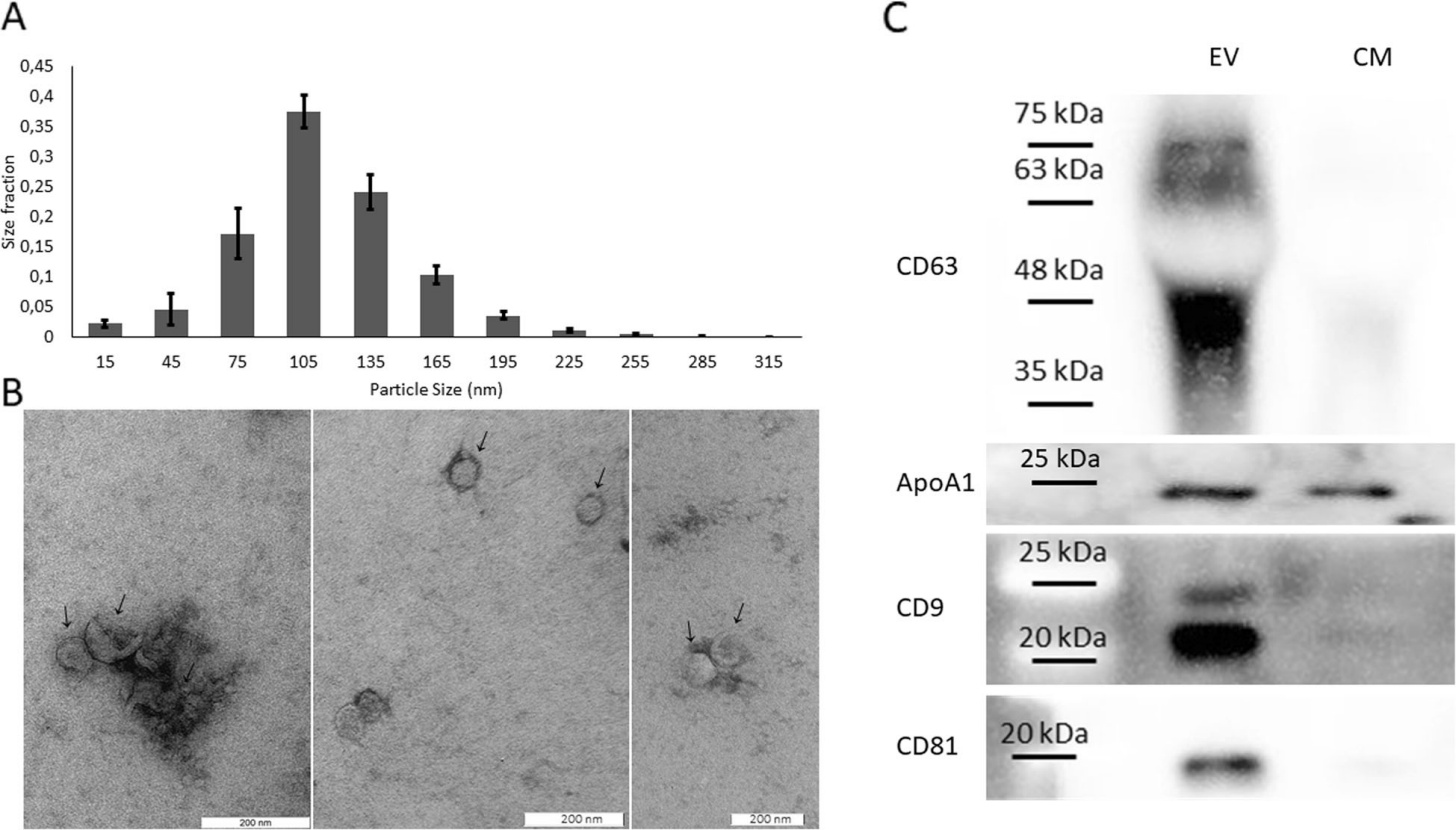
Confirmation of trophoblast spheroid derived nanoparticles as extracellular vesicles (EVs). **a** Nanoparticle tracking analysis (NTA) of trophoblast spheroid derived extracellular vesicles (EVs). Number and size profiles of EVs were analysed using ZetaView™ nanoparticle analyser. The profile exhibits a typical distribution of particles mostly less than 200 nm. Data is presented as mean ± SEM. **b** The transmission electron microscopy for EVs’ morphology. EVs visualized after staining in 2% uranyl acetate following by uranyl oxalate and methylcellulose. Scale bar = 200 nm. Classic morphological characteristics such as uniform shape, clearly discernible lipid bilayers and “cup shape” is observed. **c** Western blot analysis of trophoblast spheroid derived EVs (EV) and trophoblast spheroid conditioned media (CM). Specific protein markers for EVs (CD63, CD9 and CD81) are enriched in EV samples while negative control Apo A-I is not enriched

NTA revealed a population of particles largely under 200 nm with majority of the particles in 75–135 nm range (Fig. 4a). Electron microscopy showed uniform particles of less than 200 nm with identifiable lipid bilayer membranes, circular cross section and characteristic “cup shape” (Fig. 4b).

Western blot analysis showed that EVs’ specific protein markers CD63, CD9 and CD81 were enriched in trophoblast spheroid derived EVs compared to trophoblast spheroid conditioned culture media, while apolipoprotein A-I (a negative marker for EV) was not enriched (Fig. 4c).

### Transferred transcripts were significantly down regulated in endometrium

Endometrial cells were co-incubated with trophoblast spheroids and HEK293 spheroids in separate groups. Similar numbers of endometrial cells were supplemented with trophoblast spheroid derived EVs and HEK293 spheroid derived EV in separate groups. HEK293 spheroids and HEK293 derived EVs were used as a negative control along with untreated endometrial cells. After 24 h of co-incubation, endometrial cell RNA was analysed for the expression of candidate transcripts using qPCR.

**Fig. 5:**
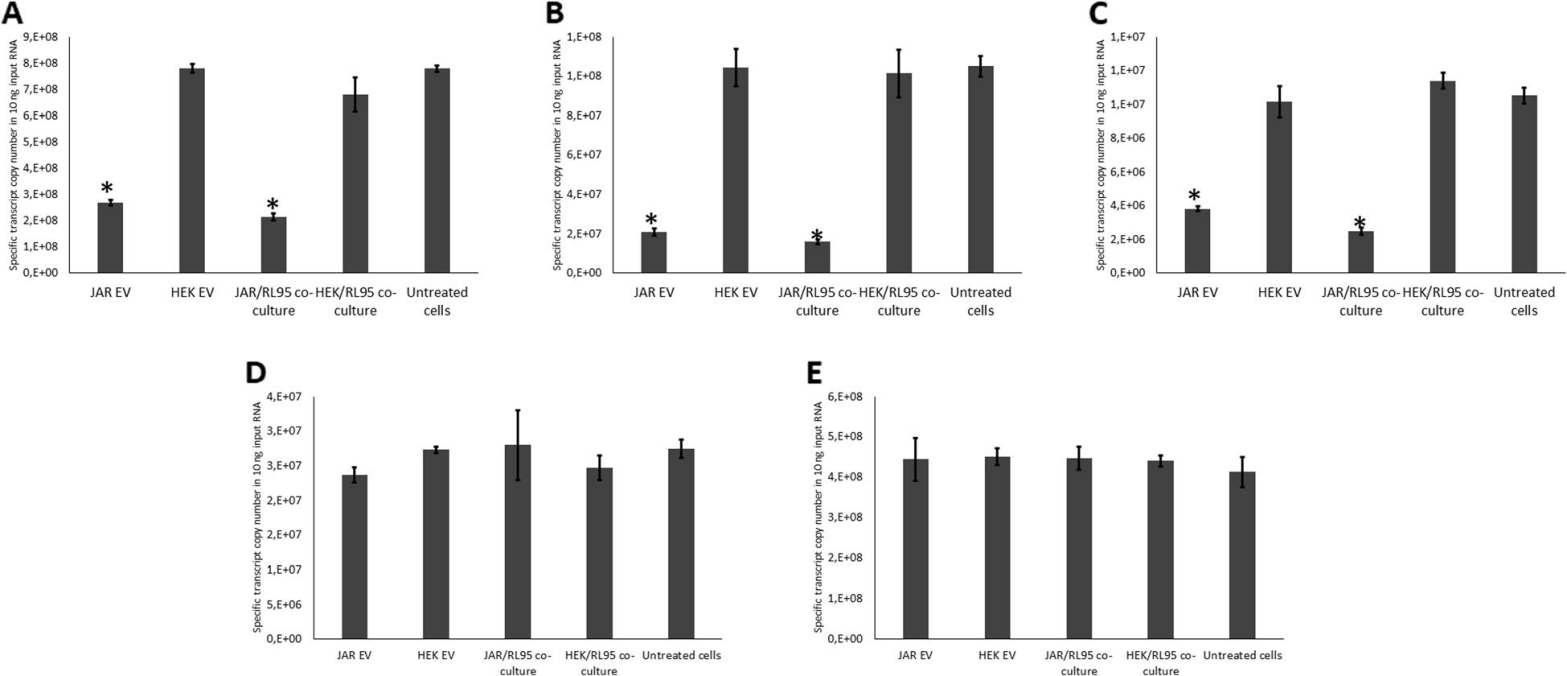
Quantification of transferred and control transcripts’ expressions in endometrial cells. Expressions of (**a**) Intronic-region of LINC00478, (**b**) Exonic region of LINC00478, (**c**) ZNF81, (**d**) beta actin and (**e**) beta-2-microglobulin in endometrial cells in co-culture with trophoblast spheroids, co-culture with HEK293 spheroids, treated with JAr derived extracellular vesicles (EVs), treated with HEK293 derived EVs and untreated control. Spheroids were co-incubated with endometrial cell monolayer for 24 h. Isolated EVs were incubated with endometrial cells for 24 h. Whole RNA of endometrial cells was quantified using qPCR for expression of transferred/control transcripts. Data is presented as mean ± SEM. (*) *p* < 0.05 vs untreated control

The three transferred transcripts showed significant down-regulation in endometrial cells co-cultured with trophoblast spheroids compared to untreated controls and endometrial cells co-cultured with HEK293 spheroids. Transferred transcripts were also significantly down-regulated in endometrial cells treated with trophoblast derived EVs compared to untreated controls and endometrial cells treated with HEK293 derived EVs (Fig. 5a, b, c). Control genes (beta-actin and beta-2-microglobulin) did not show a significant change of gene expression between the groups (Fig. 5d, e).

### Embryo derived EV/nanoparticles alter the expression of specific transcripts in endometrial cells

Conditioned media was collected from both viable and degenerating human embryos at day 3 and day 5 post IVF. EVs were isolated from conditioned and supplemented to endometrial cells. After 24 h of EV supplemented incubation, whole RNA from endometrial cells were collected and analysed for the expression of candidate genes by qPCR.

**Fig. 6:**
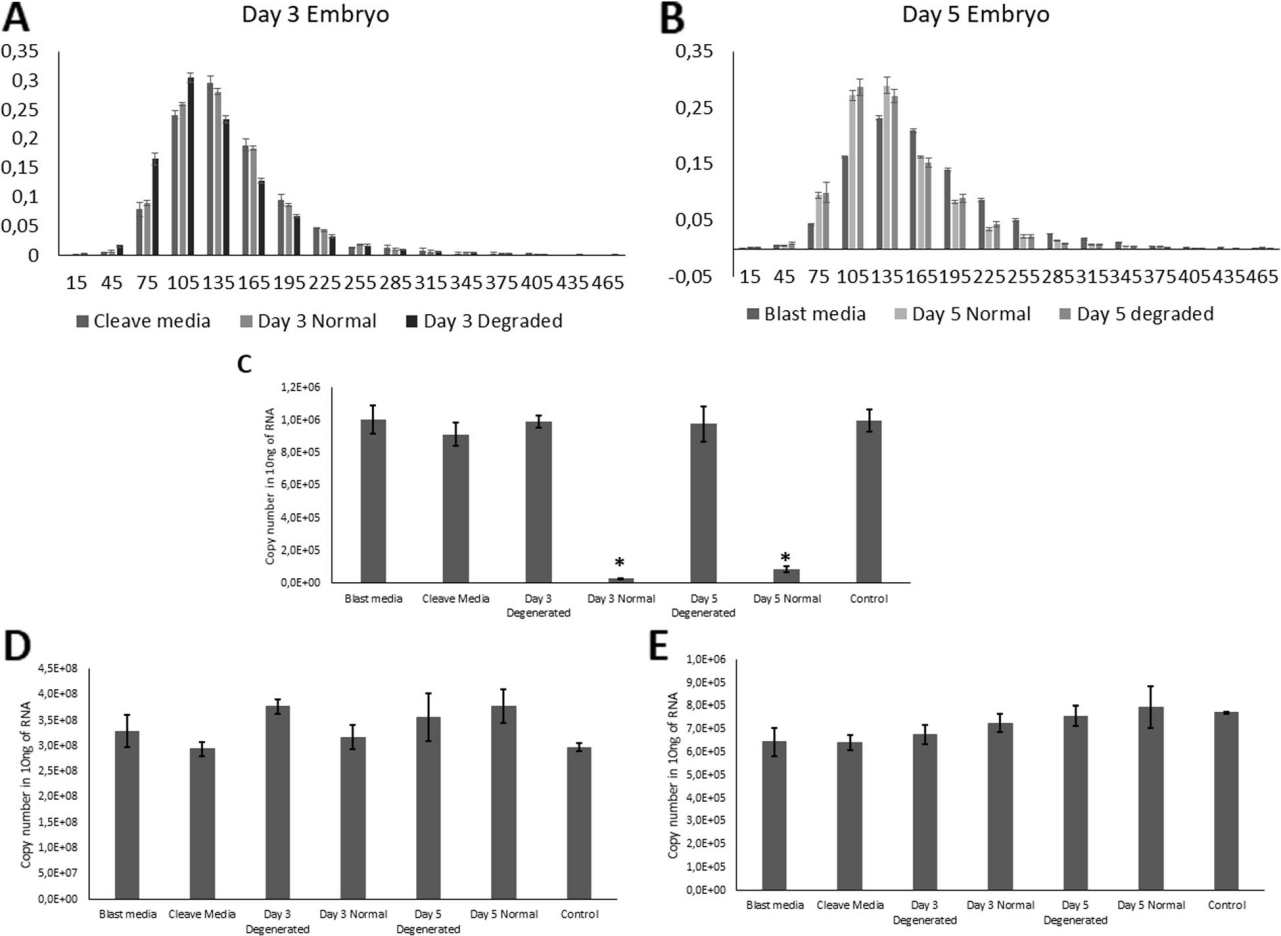
Embryo-derived extracellular vesicles (EVs) alter the expression of specific transcripts in endometrial cells. **a**, **b** Size profiles of embryo and embryo culture media derived nanoparticles strongly resemble a typical size profile of a population of comparable EVs. Gene expressions of **c** ZNF81, **d** Beta-2-microglobulin and **e** Beta actin in endometrial cells treated with human IVF day 3/5 normal/degenerating embryo-derived EVs, pure culture media derived EVs and untreated control. Isolated EVs were incubated with endometrial cells for 24 h and whole RNA of cells was quantified using qPCR. Data is presented as mean ± SEM. (*) *p* < 0.05 vs untreated control

The size profile of nanoparticles derived from embryo conditioned media (Fig. 6a, b) exhibits the characteristics of a typical EV population. EVs derived from both day 3 and 5 normal quality embryos induced a significant down-regulation of *ZNF81* transcript (Fig. 6c). EVs derived from day 3 and 5 degenerating embryos did not induce similar change in the expression of *ZNF81*. Control genes (beta-actin and beta-2-microglobulin) did not show a significant change of gene expression between the groups (Fig. 6d, e).

## Discussion

A new paradigm has arisen in the scientific literature, pointing to the transfer of genetic material, and in particular different forms of RNA as an important mediator of the process of cell-to-cell communication [37], examples of which have been documented from across diverse taxa. There is evidence of plant cells using ncRNA to communicate within and between the cells [8, 38–40]. These examples are not limited to communication between the members of one species. Inter-species and inter-kingdom communication using ncRNA is also evident. A recent example is the case of miRNAs from the parasitic plant *Cuscuta campestris* targeting host messenger RNAs in the host plants and changing the transcription profile of the host plant [41]. Plants use ncRNA to fight fungal infections by inhibiting fungal growth [42–44]. In the human context, ncRNA is also likely to play a major role in intercellular communication. A well-known example is the communication and exchange of genetic material involving cancerous cells metastasing to different tissues^39,40^. It seems that cancerous cells are capable of signalling the cells of distant tissues, resulting in the remodelling of those tissues to better support metastatic tumour growth. The signals conveyed by cancerous cells seem to be in the form of ncRNA [9, 45–47].

In nearly all these scenarios, ncRNAs seem to be transferred from one cell to another. Thereafter, the transferred material acts upon gene expression regulation in the recipient cells and changes the transcriptomic profile of them. The consequences of such communication would lead to alterations in the function and physiology of the cells, and ultimately may even result in the occurrence of disease or in the case of reproduction may affect conception and maintenance of the pregnancy. There is evidence of the exchange of miRNA between the pre-implantation embryo and the endometrium [5] and vice versa [6]. Exchanged ncRNA could perform a number of functions in the target cells. Considering the lack of immune response towards embryo, which should be identified as “non-self”, from the maternal immune system, one such function could be the modification of maternal immune response. Indeed, there are evidence of maternal immune system treating the embryo as a “temporary self” and assume “immune ignorance” [48–50]. Initiation and regulation of such unique immune response could be due to epigenetic modification caused by transferred genetic material by the developing embryo.

In the present study we used biorthogonal click chemistry to track trophoblastic RNA and its uptake by endometrial cells. Compared to other enzyme dependent labelling solutions such as 5-bromouridine (BrU), 5-iodouridine (IU), or 5-fluorouridine (FU) which rely on indirect immunofluorescence, EU has a significant advantage to be compatible to be used in Click-chemistry and downstream applications requiring affinity precipitation of labelled RNA [51]. However, the efficiency of tagging is around one nucleotide in 35, which is not significantly different from the other labelling methods [52]. Another important factor causing approximately 35% non-specifically captured unlabelled RNA in our investigation is the problems associated with RNA Recovery using affinity precipitation protocols.

In the current investigation the origins of three transcripts were identified to be transferred from embryonal to endometrial cells: an intronic-non-coding region and an exonic-coding region, originating from LINC00478, and an exonic-coding region originating from *ZNF81* gene (Additional file 1: Table S2). In the case of transcripts originating from LINC00478, Dfam v2.0 software showed that this transcript matches with *LTR7B* family (ERV1 endogenous retrovirus super family) [53]. Open reading frame prediction demonstrated that 5 kbp upstream of this region might be a considerable potential for endogenous retrovirus protein [54]. The regulatory role of endogenous retroviruses elements in development of human pre-implantation embryo has been strongly emphasized [55]. It has been demonstrated that *LTR7B* and *LTR7Y* are enriched in the eight-cell/morula and blastocyst stage embryos, respectively. *LTR7* copies can produce specific class of lncRNA [56] and in human embryonic stem cells they are involved in the regulatory network of pluripotency [57]. Specific class of ncRNA can also be produced from endogenous retrovirus ERV9, activating the transcription of erythropoiesis genes [58]. These elements can be horizontally transferred via EVs during intercellular communication. For instance, it has been confirmed that the RNA sequence of retrotransposon from human ERVs can be packaged into the EVs and transferred and spread during tumorigenesis [59]. In addition, the protein products of endogenous retroviral elements (such as envelope glycoprotein syncytin-2) are essential for early embryo and placenta development during implantation and these proteins are transferred by exosomes and are up-taken by endometrial cells [60–62].

We were not able to precipitate measurable amounts of *ZNF81* transcript from EU labelled spheroid derived EVs due to the low efficiency of EU labelled RNA capture system. It has been shown that zinc-finger protein family can cooperate with transposable elements to form an epigenetic regulatory network [63–65]. In the case of *ZNF81*, it is believed that this protein has the potential binding site for LINE elements (long interspersed nuclear elements) involved in regulation of many gene expression regulatory networks [63].

In all the three identified transferred transcripts, the endogenous expression of the same transcripts in the endometrial cells was significantly down-regulated after JAr cell or JAr cell-derived EVs’ co-culture (Fig. 5). Down-regulation of gene expression in target cells has been observed in the context of intercellular communication in different cell types [66, 67]. RNA-mediated gene expression down-regulation could be achieved using one of the several pathways, such as post-transcriptional gene silencing, co-suppression, quelling, and RNA interference (RNAi) [68]. Recent investigations have postulated that negative feedback mechanisms are utilized by lncRNA to regulate self-expression [69–71]. LincRNA are capable of increasing or decreasing self-expression or the expressions of specific target genes by interacting with chromatin-modifying complexes to modulate the epigenetic landscape of chromatin [72, 73]. Although the effect of RNA transfer on endogenous RNA down-regulation observed in the current study is likely achieved by the RNA-mediated gene expression regulation, the exact molecular mechanism remains to be discovered by the future studies. However, the possible involvement of RNA-independent mechanism cannot also be entirely excluded due to the heterogeneous nature of EV cargo. To confirm that the EV-transferred transcripts are responsible for the down-regulation of the same endogenous genes, a gene knockout trophoblast model producing EVs without particular transcripts would be essential. Such knock-out trophoblasts will produce EVs without transferred transcript and will allow detailed analysis of counterpart endogenous gene expression and protein function in endometrial cells treated with these modified EVs.

Up to this point, all of the conclusions arrived are based on data gathered using in vitro analogues of trophoblasts. We have used EVs isolated from IVF human embryo conditioned media to confirm that the observed phenomenon is common for both in vitro model and the embryo. One of the main criticisms of assisted reproduction has been its high tendency to cause multiple births. To avoid the issue, single embryo transfer is often practised. Selecting the best embryo for transfer is important in single embryo transfer procedures [74]. Until very recent past, the selection was done using morphological criteria, such as the number of blastomeres, the absence of multinucleation, early cleavage to the two-cell stage, a low percentage of cell fragments in embryos, the blastocoelic cavity expansion and the cohesiveness and number of the inner cell mass and trophectodermal cells [75, 76]. Despite of the evolution of the selection criteria for IVF embryos, the rate of live birth remains as low as 30% [77]. Protein biomarkers from culture media (soluble human leukocyte antigen-G (sHLA-G) and ubiquitin) [78, 79] and cumulus cell transcriptomic markers (cyclooxygenase 2 (COX2), steroidogenic acute regulatory protein (STAR), and pentraxin 3) have been proposed as tools for embryo selection [80–82] without major improvement in the embryo implantation rate.

EVs isolated from conditioned culture media of IVF embryos as early as on day 3 after fertilization have the potential to be used as non-invasive biomarkers for embryo selection. In the current study we provide evidence that EVs/nanoparticles isolated from embryo conditioned culture media can induce a measurable effect on endometrial cells and the effect is only seen when using conditioned media from morphologically good-quality embryos as opposed to degenerating embryos. Although the minimal requirements for EV studies require NTA, Western blot analysis of EV specific proteins and electron microscopy as per International Society for Extracellular Vesicles (ISEV) guidelines [83], due to the low number of particles isolated from single embryo culture media, Western blot analysis are currently not feasible in this context. However, with the NTA results, it could be argued that these nanoparticles are highly likely to constitute EVs. In the current study endometrial *ZNF81* expression was significantly down-regulated after EV-co-incubation originating from good-prognosis day 3/5 IVF embryos. To the contrary, the EVs from poor prognosis embryos were unable to initiate any changes of endometrial cells. We therefore suggest that with the further development, the EV-based method could be used as a non-invasive tool for selecting high-quality IVF embryos for transfer.

The dose of the EVs received by endometrial cells should also be considered as a limiting factor in the model. Multiple spheroids were used instead of one, which would be more in line with the natural environment of the embryo-maternal interface, because of the very low amount of EVs released from a single spheroid. Analysing such minuscule amounts of RNA is extremely difficult especially considering the low efficiency of labelling observed. It should also be pointed out that in vivo*,* only a few endometrial cells need to be affected by the communications from an embryo, while in the in vitro model, the effect should be seen in thousands of cells to be detected by qPCR.

## Conclusion

In conclusion, we present the evidence of non-contact transfer of embryonic RNA transcripts to endometrium in an in vitro embryo-maternal cross-talk model. RNA is taken up by the endometrial cells and the expression of endogenous transcripts are altered as a result. The effect can be seen in endometrial cells treated with EVs derived from IVF embryos suggesting that the RNA is transferred through EVs. EVs derived from human IVF embryos also have the potential to change the endometrial transcripts. Interestingly, only good-prognosis embryos induced the observed effect while degenerated embryos failed to initiate any changes. Physiological effects of observed transcript changes are still not fully understood and require follow-up studies. However, with further development, these observations could be taken as a step further in the path towards understanding the first language of communication between mother and embryo.

## Supplementary information

**Additional file 1.** Data pertaining to the identified putatively transferred transcripts, specific primers used in qPCR and sequences of transferred transcripts.

## Data Availability

The sequencing data has been uploaded to the NCBI SRA repository (www.ncbi.nlm.nih.gov/sra) under the accession number PRJNA527834.

## Abbreviations

BrU: 5-bromouridine
DE: Differential expression
EU: 5-ethynyl uridine
EV: Extracellular vesicles
FU: 5-fluorouridine
ICSI: Intracytoplasmic sperm injection
IU: 5-iodouridine
IVF: In vitro fertilization
lincRNA: Long intergenic/intervening RNA
LINE: Long interspersed nuclear elements
lncRNA: Long non-coding RNA
miRNA: Micro RNA
ncRNA: Noncoding RNA
NTA: Nanoparticle tracking analysis
RNAi: RNA interference
