# Supplementary material for: Specific trophoblast transcripts transferred by extracellular vesicles affect gene expression in endometrial epithelial cells and may have a role in embryo-maternal crosstalk
Source: Cell Commun Signal. 2019 Nov 14;17:146. doi: 10.1186/s12964-019-0448-x (PMC6854687; doi:10.1186/s12964-019-0448-x)
Supplement: Supplementary file 1 — Additional file 1. Data pertaining to the identified putatively transferred transcripts, specific primers used in qPCR and sequences of transferred transcripts. [file 12964_2019_448_MOESM1_ESM.pdf]

Table.1: The table of primers and sequence information

Transcript Name	Primer Sequence (5‘-3‘)
ZNF81	Forward primer: TGATACAGAAGACTTGAGATT<br>Reverse primer: TCACAAAGTATTCACATTACC
Exonic LINC00478	Forward primer: TCAAGTTCAGTGTTTGGTTAA<br>Reverse primer: GGCAGAATCGTGAATAGC
Intronic LINC00478	Forward primer: AACAGGTCACAATGGTGGAATG<br>Reverse primer: TGAAGCAACTGAAGATCCACAA
Beta-2-microglobulin	Forward primer: CGGGCATTCCTGAAGCTGA<br>Reverse primer: TGGAGTACGCTGGATAGCCT
Beta-actin	Forward primer: GTGCGCCGTTCCGAAAGT<br>Reverse primer: ATCATCCATGGTGAGCTGGCG
Synthetic RNA Spike-in (100 bp from Isopenicillin N-CoA synthetase)	Spike-in Forward primer: TACTGCATCCCGCTCTAC<br>Spike-in Reverse primer: CGCTCATCAAGTCGTTCA<br>Spike-in RNA sequence: UUGGGCAGAAACCGGGCCCCAACGGUGACCGCACCUACU ACUGCAUCCCGCUCUACCACGGAACGGGGGGCAUCGCGGCCAUGAACGACUUGAUGAGCGG

Table 1. Primer and spike-in RNA sequences.

Specific primers used for qPCR analysis of transferred/control transcripts. Primers were designed using Beacon Designer™ (PREMIER Biosoft International, Palo Alto, USA). Primer efficiency was measured using cDNA gradient method. Efficiency in the chosen temperature profile was between 98.7% and 99.2%.

Table.2: Putatively transferred transcripts.

	Gene	logFC	logCPM	LR	PValue	FDR
1	MUC4	4,962	1,84E+00	2,76E+01	1,50E-07	1,64E-04
2	MUC3A	4,09E+00	3,69E+00	2,73E+01	1,72E-07	1,64E-04
3	MUC16	3,59E+00	3,57E+00	2,20E+01	2,68E-06	1,12E-03
4	MUC12	3,40E+00	2,98E+00	1,93E+01	1,12E-05	3,41E-03
5	ZNF81	4,43E+00	-2,96E-01	1,75E+01	2,93E-05	6,97E-03
6	RRAGB	4,22E+00	-7,88E-02	1,69E+01	3,87E-05	8,32E-03
7	MT-TW	2,84E+00	3,89E+00	1,48E+01	1,21E-04	2,13E-02
8	Z95704,5	3,72E+00	1,20E-01	1,42E+01	1,67E-04	2,48E-02
9	MT-TS1	2,67E+00	5,02E+00	1,31E+01	2,91E-04	3,29E-02
10	ITGAE	3,54E+00	9,15E-02	1,29E+01	3,30E-04	3,48E-02
11	RP11-357C3,3	2,98E+00	1,85E+00	1,29E+01	3,33E-04	3,48E-02
12	TMEM154	3,45E+00	4,12E-01	1,28E+01	3,40E-04	3,48E-02
13	CASP14	3,35E+00	4,68E-01	1,22E+01	4,87E-04	4,33E-02
14	ZNF765	3,31E+00	5,09E-01	1,20E+01	5,26E-04	4,45E-02
15	LINC00478	3,38E+00	-1,14E-01	1,18E+01	5,87E-04	4,69E-02
16	MT-TQ	2,56E+00	7,00E+00	1,16E+01	6,63E-04	4,85E-02
17	ANKRD44	3,22E+00	7,80E-01	1,15E+01	6,78E-04	4,85E-02
18	ZBED3-AS1	3,29E+00	-1,13E-01	1,15E+01	6,98E-04	4,85E-02

Table.2: Putatively transferred transcripts.

The 18 putatively transferred transcripts identified using glmLRT function of edgeR package. RNA sequencing was carried out using RNA affinity precipitated from endometrial cells co-incubated for 24 hours with EU labeled trophoblast spheroids. Endometrial cells co-incubated with a similar number of unlabeled trophoblast spheroids were used as a negative control.

Table 3: Sequences of transfered transcripts

Transcript	Sanger Sequence
ZNF81	TGATACAGAAGACTTGAGATTCTGGATTGGAGCTTGATGCCACAATTTTGGATGAGAAATTTGGAGGTCCTGGAATAGG
Exonic LINC00478	TCAAGTTCAGTGTTTGGTTAAAATACATACTCAGTAAATGGTAGCTATTATTGTCTTAGTTTAAGTTATTGCAAGCATTAAAATTAAATGTTTAGCTACAGACTCAATCCAGTTTTAATGTCATTGTGTTAATAAGGCCTCTTAACATTGAAGCAACAAAGA
Intronic LINC00478	AACAGGTCACAATGGTGGAATGTCGTCAGCTAAGGCAGGACCTGGCTATTTGCACTTCTTTTGTGGATCTTCAGTTGCTTCA

Table.3: Sequences of transferred transcripts.

Expression of transferred transcripts were quantified using qPCR. Products of qPCR were purified using column purification (MinElute PCR Purification Kit, Qiagen, No 28004) and sequenced using Sanger method.
